# Cross-sectional study of the associations between circulating vitamin D concentrations and insulin resistance in children aged 9–10 years of South Asian, black African Caribbean and white European origins

**DOI:** 10.1136/jech-2023-220626

**Published:** 2023-12-11

**Authors:** Angela Donin, Claire M Nightingale, Naveed Sattar, William D Fraser, Chris G Owen, Derek G Cook, Peter H Whincup

**Affiliations:** 1 Population Health Research Institute, St George's University of London, London, UK; 2 Institute of Cardiovascular & Medical Sciences, University of Glasgow, Glasgow, UK; 3 Faculty of Medicine and Health Sciences, University of East Anglia, Norwich, UK; 4 Norfolk and Norwich University Hospitals NHS Foundation Trust, Norwich, UK

**Keywords:** CHILD HEALTH, EPIDEMIOLOGY, NUTRITION, DIABETES MELLITUS

## Abstract

**Background:**

Lower circulating vitamin D 25-hydroxyvitamin D (25(OH)D) concentrations are associated with higher type 2 diabetes risk in adults, although causality remains uncertain. However, associations between 25(OH)D and type 2 diabetes risk markers in children have been little studied, particularly in ethnic minority populations. We examined whether 25(OH)D concentrations were associated with insulin resistance in children and whether lower 25(OH)D concentrations in South Asians and black African Caribbeans could contribute to their higher insulin resistance.

**Methods:**

Cross-sectional study of 4650 UK primary school children aged 9–10 years of predominantly South Asian, black African Caribbean and white European ethnicity. Children had fasting blood measurements of circulating 25(OH)D metabolite concentrations, insulin and glucose.

**Results:**

Lower 25(OH)D concentrations were observed in girls, South Asians and black African Caribbeans. In analyses adjusted for age, sex, month, ethnic group and school, circulating 25(OH)D was inversely associated with fasting insulin (−0.38%, 95% CI −0.49% to −0.27%), homoeostasis model assessment (HOMA) insulin resistance (−0.39%, 95% CI −0.50% to −0.28%) and fasting glucose (−0.03%, 95% CI −0.05% to –0.02%) per nmol/L increase in 25(OH)D; associations did not differ between ethnic groups. Ethnic differences in fasting insulin and HOMA insulin resistance (higher among South Asian and black African Caribbeans) were reduced by >40% after adjustment for circulating 25(OH)D concentrations.

**Conclusion:**

Circulating vitamin D was inversely associated with insulin resistance in all ethnic groups; higher insulin resistance in South Asian and black African children were partly explained by their lower vitamin D levels. Whether vitamin D supplementation can reduce emerging type 2 diabetes risk needs further evaluation.

**WHAT IS ALREADY KNOWN ON THIS TOPIC**

- Observational studies have shown that lower circulating vitamin D concentrations are associated with increased risk of type 2 diabetes, though the results of vitamin D supplement trials have been inconsistent.
- Baseline vitamin D status may be particularly important as some studies have suggested that participants with low baseline vitamin D concentrations may be more likely to show a reduction in insulin resistance than replete participants. This could be particularly relevant to ethnic minority groups with low vitamin D concentrations and high risk of type 2 diabetes.

**WHAT THIS STUDY ADDS**

- This study examined the association between circulating vitamin D concentrations and insulin resistance in South Asian, black African Caribbean and white European children.
- This study highlights the low average vitamin D concentrations in UK primary school children (average concentrations below vitamin D sufficiency concentrations of >50 nmol/L), particularly in children of South Asian and black African Caribbean origins.
- Our findings indicate strong inverse associations between vitamin D concentrations and insulin resistance in 9–10 years, with similar associations present in children of different ethnic origins.
- The higher insulin resistance observed in children of South Asian and black African Caribbean origins, compared with white European children, could be at least partly explained by their lower vitamin D status (by ~40%).

**HOW THIS STUDY MIGHT AFFECT RESEARCH, PRACTICE OR POLICY**

- The inverse association between childhood vitamin D and insulin resistance shown in this study in all ethnic groups raises the possibility that improving vitamin D status could reduce longer-term emerging type 2 diabetes risk. This could be especially important in South Asian and black African children, shown in this study to have higher insulin resistance and lower vitamin D status.
- Further research is needed to establish whether vitamin D supplementation in childhood reduces insulin resistance (as well as improving bone health), perhaps especially in South Asian and black African Caribbean children.

## Introduction

Vitamin D is a secosteroid hormone with a central role in bone homoeostasis.^1^ Total vitamin D comprises two forms, vitamin D_3_ (cholecalciferol) and vitamin D_2_ (ergocalciferol), which are converted in the liver to 25-hydroxyvitamin D [25(OH)D]; circulating levels of 25(OH)D and its separate forms 25(OH)D_2_ and 25(OH)D_3_ are used to assess vitamin D status. In the UK, vitamin D deficiency is defined as circulating 25(OH)D concentrations <25 nmol/L, vitamin D insufficiency as circulating 25(OH)D concentrations between 25 nmol/L and 50 nmol/L.^2^ It is recommended to consume 10 µg vitamin D a day, throughout the year, to maintain serum 25(OH)D concentrations above those representing deficiency.^3^ However, almost one in five UK adults and children are vitamin D deficient,^4 5^ with much higher proportions of UK South Asian and black African Caribbean adults reported to be deficient (~35% and 53%, respectively)^6^; similar patterns have been reported in Europe.^7^

Alongside the known skeletal functions of vitamin D, it has been suggested that having low total 25(OH)D concentrations increases risks of cardiovascular disease^8^ and type 2 diabetes,^9^ possibly through the multifunctional roles of 25(OH)D in myocardial contractility, β-cell function and insulin production.^10^ In adults, longitudinal studies have demonstrated strong and consistent associations between low circulating 25(OH)D and higher risks of type 2 diabetes^9 11^ and insulin resistance.^12^ However, the results of Mendelian randomisation studies, though providing some support for a causal association between low 25(OH)D concentration and higher type 2 diabetes risk^13^ have not done so consistently.^14–16^ Randomised controlled trials (RCT) of vitamin D supplementation in adults have mostly been inconclusive.^17 18^ However, a meta-analysis of RCTs of vitamin D supplementation in adults with pre-diabetes reported a reduction in type 2 diabetes incidence by up to 15%,^19^ while in adults with diabetes, vitamin D supplementation reduced fasting glucose and insulin resistance.^17^ In addition, a meta-analysis of predominantly small trials in children has indicated that vitamin D supplementation, particularly in those who were vitamin D deficient at baseline, could reduce fasting blood glucose.^20^

These findings suggest that studies investigating the association between vitamin D and type 2 diabetes risk need to focus more strongly on populations with low vitamin D status.^20 21^ In the UK, both adults and children of South Asian and black African and Caribbean origins typically have much lower 25(OH)D concentrations and are also at high risk of type 2 diabetes.^22^ Some evidence suggests that the associations between 25(OH)D and type 2 diabetes risk markers may be modified by ethnicity, particularly in US adults,^23^ in whom associations between 25(OH)D and markers of glycaemia have been reported in non-Hispanic whites and Mexican-Americans but not in non-Hispanic blacks.^23^ Suggested mechanisms include differences in vitamin D/calcium and PTH homoeostasis, differences in levels of vitamin D binding protein and potential genetic components.^23^ We report here on the cross-sectional associations between circulating 25(OH)D concentrations and type 2 diabetes risk markers (particularly fasting insulin and homoeostasis model assessment (HOMA) insulin resistance) in a study of UK children of South Asian, black African and Caribbean, and white European origin; we also examine the extent to which lower 25(OH)D concentrations in South Asian and black African Caribbean children can account for their higher insulin resistance. Data are presented separately for 25(OH)D_2_ and 25(OH)D_3_ concentrations, as their associations may vary in strength and direction.^24^

## Methods

The Child Heart And health Study in England (CHASE) was a cross-sectional, school-based study of risk markers for type 2 diabetes and cardiovascular disease in approximately 5000 children of South Asian, black African and Caribbean, and white European origin. This study has been described in detail elsewhere.^25^ A stratified random sample of 200 state primary schools was selected in London, Birmingham and Leicester, three cities including more than two-thirds of UK ethnic minority children. Half of schools included a high proportion of South Asian children, stratified by ethnic subgroup (Indian, Pakistani, Bangladeshi) and half a high proportion of black African children, stratified by ethnic subgroup (black African, black Caribbean). All schools selected also included between 15% and 50% white European children, allowing ethnic comparisons with white European children to be made on a within-school basis in every school. All year 5 pupils (aged 9–10 years) in participating primary schools were invited to take part.

### Blood measurements and anthropometry

A single survey team including three trained research nurses and a support fieldworker carried out all survey measurements during school terms between October 2004 and February 2007 (no data were collected during August holiday periods). Measurements of height, weight and bioelectrical impedance (Bodystat) were recorded. Fat mass index derived from bioelectrical impedance was used as the principal marker of body fat, as it provided valid measurements of body fat in this multiethnic population, in contrast with body mass index, which yielded biased results.^26^ Children provided blood samples for the measurement of all blood markers following an overnight fast (~12 hours). Assay methods for insulin, glucose andglycated haemoglobin (HbA1c) have been described elsewhere.^25^ The HOMA equations were used to provide an estimate of insulin resistance (HOMA-IR).^27^ Children provided a sample of saliva, which was measured with a gas-liquid chromatography method (detection limit 0.1 ng/mL) to determine levels of cotinine. 25(OH)D_2_ and 25(OH)D_3_ and the deuterated internal standard were measured separately after extraction from EDTA plasma samples (stored at −70°C since collection), and the deuterated internal standard were extracted from serum samples, after protein precipitation, using Isolute C18 solid-phase extraction cartridges. Potential interfering compounds were removed by initial elution with 50% methanol followed by elution of the vitamins using 10% tetrahydrofuran in acetonitrile. Dried extracts were reconstituted before injection into a HPLC tandem mass spectrometer in the multiple reaction mode. The multiple reaction mode transitions (mass to charge ratio) were 413.2 greater than 395.3, 401.1 greater than 383.3, and 407.5 greater than 107.2 for 25(OH)D_2_, 25(OH)D_3_, and hexadeuterated (OH)D_3_, respectively. Coefficients of variation for the assay were less than 10% across a working range of 1 to 250 ng/mL for both 25(OH)D_2_ and 25(OH)D_3_. Total 25(OH)D was determined from the sum of 25(OH)D_2_ and 25(OH)D_3_.

### Ethnicity and socioeconomic status

Ethnicity of the child was categorised using self-defined ethnicity provided for both parents in a study questionnaire or by using parental information on the ethnicity of the child provided by the school. In both cases, ethnicity categories were identical to those used in the 2001 UK Census. In a small number of participants for whom this information was not available (1%), a child-defined place of origin of parents and grandparents was used, cross-checked with the observer-defined ethnic appearance of the child. In the present analyses, ‘white European’ included children whose ethnic origin was defined as ‘white British’, ‘white Irish’ and ‘white European’ (or a combination of these) and excluded ‘white other’. ‘South Asian’ includes ‘Indian’, ‘Pakistani’, ‘Bangladeshi’ and ‘Sri Lankan’ (or a combination of these). ‘Black African Caribbean’ included ‘black African’, ‘black Caribbean’, ‘black British’ and ‘black other’ (or a combination of these). The ‘other’ ethnic group includes all other categories of individual and mixed ethnic origins. White European, South Asian and black African Caribbean categories were used for the main analyses in order to compare major ethnic groups with sufficient statistical power and precision, which would be lacking in analyses based on smaller ethnic subgroups. Parents and children provided information on parental occupation, which was then coded using the National Statistics-Socioeconomic Classification,^28^ resulting in the following classifications: managerial/professional, intermediate, routine/manual and economically inactive (referring to people who were currently unemployed, whether or not they are seeking work).

### Statistical analysis

Statistical analyses were carried out using STATA/SE software (STATA/SE V.17 for Windows, StataCorp). All variable distributions were checked (distributions presented in online supplemental figure 1) and the assumptions underlying linear regression modelling were tested. Vitamin D values lower than the detection limits (0.1 nmol/L for both D2 and D3) were assigned a notional value equal to the detection limit divided by the square root of 2 (0.1/1.41 nmol/L in this case).^29^ Multilevel linear regression models were used to provide adjusted means (adjusted to the average level of each variable in the model, so that the values are close to the observed data) and to quantify the associations between risk markers for type 2 diabetes and either 25(OH)D, 25(OH)D_2_ or 25(OH)D_3_, expressed as percentage differences and 95% CIs per 1 nmol/L increase in vitamin D concentration,. All analyses were adjusted for sex, age in quartiles, ethnic group and month as fixed effects; school was fitted as a random effect to allow for the clustering of children within schools. Supplementary analysis included additional adjustments for fat mass index and socioeconomic status. To test formally whether the associations between 25(OH)D and type 2 diabetes risk markers varied by ethnicity, an interaction term was fitted between 25(OH)D (or 25(OH)D_2_ or 25(OH)D_3_) and ethnicity. In further multilevel linear regression analyses, the potential impact of 25(OH)D on ethnic differences in fasting insulin and HOMA-IR between both South Asians and black African Caribbean children compared with white European children was investigated. The changes in percentage differences in these risk markers following standard adjustments (age in quartiles, sex, month and school as a random effect) from those following additional adjustment for total 25(OH)D are reported; no interaction term was fitted here, as the associations between 25(OH)D and these insulin resistance variables were similar in all ethnic groups.

10.1136/jech-2023-220626.supp1 Supplementary data

## Results

### Participants and key variables

Of 8641 children invited to participate in CHASE, 5887 (68%) took part and of those, 4650 (54%) provided fasting blood samples, had complete risk marker measurements and data on circulating 25(OH)D_2_ and 25(OH)D_3_ concentrations. On average, approximately 23 children participated in each of the 200 schools (mean 23.3, median 21). The mean age was 9.96 years (95% reference range 9.30–10.62) and 52% were girls, with approximately equal numbers of white European, South Asian, black African Caribbean and children of other or mixed ethnic origins (1117, 1275, 1176 and 1082, respectively). 25(OH)D_2_ concentrations were below the level of detection in 1041 children (22%) and were adjusted as described in the Methods section.

Total 25(OH)D concentrations were more strongly correlated with 25(OH)D_3_ (r=0.96) than with 25(OH)D_2_ (r=0.24). Mean concentrations of 25(OH)D and 25(OH)D_3_ but not 25(OH)D_2_ were lower in girls than in boys (p<0.0001) and in children of South Asian and to a lesser extent black African Caribbean and children of other ethnic origins than in white Europeans (p<0.0001) (table 1). Type 2 diabetes risk markers showed marked differences by sex and ethnicity (table 1). Girls had higher levels of fasting insulin, HOMA-IR, triglyceride and fat mass index and lower levels of fasting glucose, 25(OH)D and 25(OH)D_3_ compared with boys. South Asians and to a lesser extent black African Caribbean children had higher levels of fasting insulin, HOMA-IR and HbA1c than white Europeans (all p<0.0001), as previously reported.^25^ Analyses by quartiles of vitamin D showed similar patterns of association with gender and ethnicity and between higher vitamin D levels and higher socioeconomic status (online supplemental table 1).

**Table 1:** Risk markers for type 2 diabetes and vitamin D concentrations by gender and ethnic group

Outcome	Boys (n=2240)	Girls (n=2410)	P value (gender difference)	White European (n=1117)	South Asian (n=1275)	Black African Caribbean (n=1176)	Other (n=1082)	P value (ethnic difference)
	Mean (SD/GSD)	Mean (SD/GSD)		Mean (SD/GSD)	Mean (SD/GSD)	Mean (SD/GSD)	Mean (SD/GSD	
Insulin (mU/L)*	6.49 (1.88)	8.13 (1.89)	<0.0001	6.20 (1.80)	8.09 (1.89)	7.59 (2.00)	7.31 (0.62)	<0.0001
HOMA IR*	0.82 (1.87)	1.02 (1.88)	<0.0001	0.78 (1.80)	1.02 (1.87)	0.95 (1.97)	0.92 (0.62)	<0.0001
Glucose (mmol/L)*	4.56 (1.08)	4.48 (1.08)	<0.0001	4.51 (1.08)	4.54 (1.08)	4.50 (1.09)	4.51 (0.08)	0.01
HbA1c (%)*	5.23 (1.07)	5.25 (1.06)	0.19	5.18 (1.05)	5.29 (1.06)	5.27 (1.08)	5.22 (0.06)	<0.0001
Triglyceride (mmol/L)*	0.76 (1.50)	0.85 (1.46)	<0.0001	0.80 (1.48)	0.90 (1.50)	0.71 (1.43)	0.82 (0.38)	<0.0001
Fat mass index (kg/m^5^)*	1.91 (1.49)	2.17 (1.46)	<0.0001	2.00 (1.50)	2.15 (1.44)	1.87 (1.49)	2.12 (0.39)	<0.0001
25(OH)D (nmol/L)*	38.5 (1.7)	35.0 (1.7)	<0.0001	54.0 (1.5)	27.4 (1.7)	32.7 (1.6)	39.2 (0.5)	<0.0001
25(OH)D_2_ (nmol/L)	6.5 (5.9)	6.3 (5.5)	0.29	6.5 (5.5)	6.5 (5.9)	6.1 (5.4)	6.6 (5.8)	0.09
25(OH)D_3_ (nmol/L)*	31.4 (1.8)	28.0 (1.9)	<0.0001	47.0 (1.5)	20.6 (1.8)	26.2 (1.7)	32.2 (0.6)	<0.0001

All models are adjusted for age, sex, ethnic group, month and school (random effect).

*Geometric mean and geometric SD are shown for log transformed variables.

GSD, geometric SD; HbA1c, glycated haemoglobin; HOMA-IR, homoeostasis model assessment-insulin resistance; 25(OH)D, 25-hydroxyvitamin D.

### Associations between 25(OH)D concentrations and type 2 diabetes risk markers

**Figure 1:**
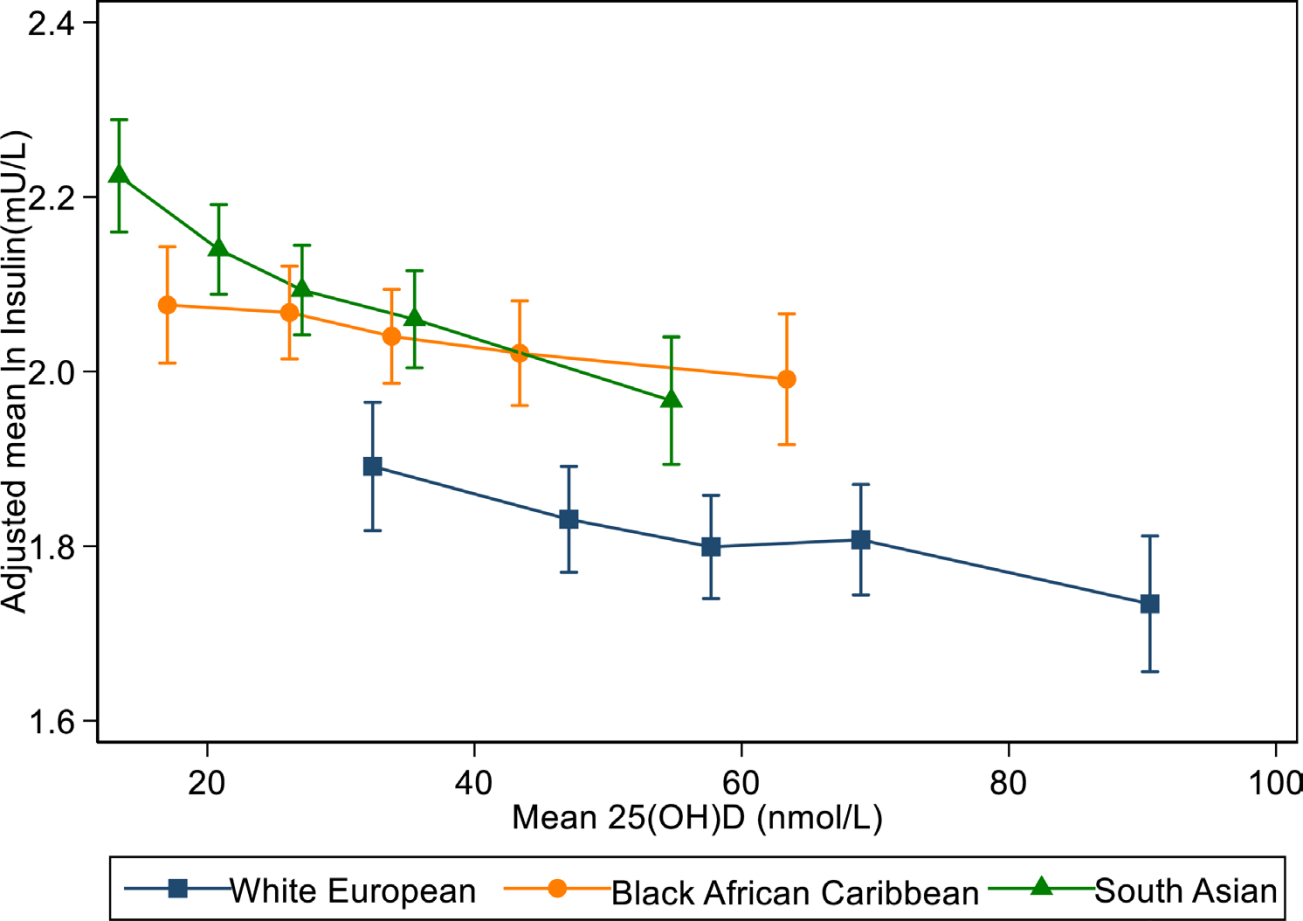
Associations between fasting insulin and total 25(OH)D: by ethnic group. Geometric means for insulin are adjusted for age, sex, month and school (random effect), presented by quintile of total 25(OH)D and ethnic group. 25(OH)D, 25-hydroxyvitamin D.

The associations between total 25(OH)D and type 2 diabetes risk markers (expressed as percentage differences in outcome variables per 1 nmol/L higher 25(OH)D concentrations) in analyses adjusted for age, sex, month, ethnic group and school, are shown for all children and separately for white Europeans, South Asians and black African Caribbeans (table 2 and figure 1, with ‘other’ ethnicity in online supplemental table 2). Total 25(OH)D showed strong graded inverse associations with fasting insulin, HOMA-IR, fasting glucose and fat mass index for all the children combined; no overall associations were apparent for HbA1c and triglycerides. There were no appreciable differences in the associations between circulating 25(OH)D, fasting insulin and HOMA-IR between males and females (data not presented). Moreover, the associations for fasting insulin, HOMA-IR and fasting glucose were found to be very similar across ethnic groups with no evidence of interaction with ethnicity (p>0.05), fat mass index, however, showed no association with total 25(OH)D in children of black African Caribbean origins (p>0.05). Inconsistencies in the patterns of association between 25(OH)D, HbA1c and triglycerides in specific ethnic groups were given limited emphasis, given the absence of clear associations in the whole childhood population.

**Table 2:** Associations between risk markers for type 2 diabetes and vitamin 25(OH)D (per nmol/L increase): by ethnic group

Outcome	Percentage difference in outcome per nmol/L increase in 25(OH)D (95% CI), p value							
	All children (n=4650)		White European (n=1117)		South Asian (n=1275)		Black African Caribbean (n=1176)	
	Differences (95% CIs)	P value (difference)	Differences (95% CIs)	P value (difference)	Differences (95% CIs)	P value (difference)	Differences (95% CIs)	P value (difference)
Insulin (mU/L)	−0.38 (−0.49 to 0.27)	<0.0001	−0.33 (−0.51 to 0.15)	<0.001	−0.58 (−0.81 to 0.35)	<0.0001	−0.30 (−0.51 to 0.09)	0.005
HOMA IR	−0.39 (−0.50 to 0.28)	<0.0001	−0.34 (−0.52 to 0.16)	<0.001	−0.57 (−0.80 to 0.34)	<0.0001	−0.30 (−0.51 to 0.09)	0.006
Glucose (mmol/L)	−0.03 (−0.05 to 0.02)	<0.0001	−0.03 (−0.05 to 0.01)	0.01	−0.02 (−0.05 to 0.01)	0.11	−0.03 (−0.06 to 0.01)	0.01
HbA1c (%)	0.00 (−0.01 to 0.01)	0.62	0.02 (0.00 to 0.03)	0.10	0.01 (−0.02 to 0.03)	0.55	−0.04 (−0.06 to 0.01)	0.001
Triglyceride (mmol/L)	−0.01 (−0.08 to 0.06)	0.86	−0.12 (−0.23 to 0.00)	0.04	−0.03 (−0.17 to 0.11)	0.66	0.11 (−0.02 to 0.24)	0.11
Fat mass index (kg/m^5^)	−0.14 (−0.20 to 0.07)	<0.001	−0.15 (−0.26 to 0.04)	0.01	−0.28 (−0.43 to 0.14)	<0.0001	−0.03 (−0.16 to 0.10)	to 0.68

All models adjust for age, sex, month, an interaction between ethnic group and 25(OH)D, and school (random effect).

P values for interaction between ethnicity and 25(OH)D were: insulin p=0.08, HOMA IR p=0.09, glucose p=0.96, HbA1c p<0.001, triglyceride p=0.02, fat mass index p=0.01.

NB: The total number includes the ‘other’ ethnic group which is not presented here.

HbA1c, glycated haemoglobin; HOMA-IR, insulin resistance-insulin resistance; 25(OH)D, 25(OH)D.

Associations between the 25(OH)D_2_ and 25(OH)D*_3_* forms of vitamin D and type 2 diabetes risk markers were examined separately, both overall and by ethnic group (table 3, online supplemental figure 2 and table 2 for ‘other’ ethnic group). Both forms of vitamin D had similar inverse associations with fasting insulin and HOMA-IR to those observed for total 25(OH)D in all children. Associations with 25(OH)D_3_ and glucose, HbA1c, triglycerides and fat mass index were broadly consistent with those observed for total 25(OH)D, while associations 25(OH)D_2_, both in the whole study population and in specific ethnic groups, were less consistent. When these analyses were carried out per IQR increase of each form of vitamin D (online supplemental file 1) it was clear that the relative strengths of the inverse associations between vitamin D, fasting insulin, HOMA-IR and glucose were much larger for total 25(OH)D and 25(OH)D_3_ than for 25(OH)D_2_. Additional adjustment of the analyses for fat mass index and socioeconomic status made no material difference to the analyses in table 2 (online supplemental table 3).

**Table 3:** Associations between risk markers for type 2 diabetes and cardiovascular disease and vitamin 25(OH)D_2_ and 25(OH)D_3_ (per nmol/L increase): by ethnic group

Outcome	Percentage difference in outcome per nmol/L increase in 25(OH)D_2_ (95% CI), p value							
	All n=4650)		White European (n=1117)		South Asian (n=1275)		Black African Caribbean (n=1176)	
	Differences (95% CIs)	P value	Differences (95% CIs)	P value	Differences (95% CIs)	P value	Differences (95% CIs)	P value
Insulin (mU/L)	−0.39 (−0.74 to 0.03)	0.03	0.15 (−0.52 to 0.83)	0.66	−0.22 (−0.82 to 0.39)	0.48	−1.49 (−2.15 to 0.83)	<0.0001
HOMA IR	−0.39 (−0.74 to 0.04)	0.03	0.12 (−0.54 to 0.79)	0.72	−0.20 (−0.79 to 0.40)	0.52	−1.40 (−2.05 to 0.74)	<0.0001
Glucose (mmol/L)	0.00 (−0.04 to 0.05)	0.94	0.06 (−0.02 to 0.15)	0.14	−0.02 (−0.09 to 0.06)	0.68	−0.02 (−0.11 to 0.06)	0.61
HbA1c (%)	0.05 (0.02 to 0.09)	0.003	0.06 (−0.01 to 0.13)	0.08	0.05 (−0.01 to 0.11)	0.11	0.03 (−0.04 to 0.09)	0.42
Triglyceride (mmol/L)	0.77 (0.55 to 0.99)	<0.0001	0.93 (0.52 to 1.35)	<0.0001	0.84 (0.47 to 1.21)	<0.0001	0.27 (−0.15 to 0.68)	0.21
Fat mass index (kg/m^5^)	0.24 (0.02 to 0.45)	0.03	0.41 (−0.01 to 0.83)	0.05	−0.14 (−0.51 to 0.23)	0.47	0.16 (−0.25 to 0.58)	0.44
	Percentage difference in outcome per nmol/L increase in 25(OH)D_3_ (95% CI), p value							
Insulin (mU/L)	−0.38 (−0.49 to 0.26)	<0.0001	−0.35 (−0.54 to 0.17)	<0.001	−0.65 (−0.90 to 0.40)	<0.0001	−0.17 (−0.39 to 0.05)	0.13
HOMA IR	−0.38 (−0.50 to 0.27)	<0.0001	−0.37 (−0.55 to 0.18)	<0.0001	−0.65 (−0.89 to 0.40)	<0.0001	−0.18 (−0.40 to 0.04)	0.11
Glucose (mmol/L)	−0.04 (−0.05 to 0.02)	<0.0001	−0.04 (−0.06 to 0.01)	0.003	−0.03 (−0.06 to 0.01)	0.11	−0.03 (−0.06 to 0.01)	0.01
HbA1c (%)	−0.01 (−0.02 to 0.00)	0.13	0.01 (−0.01 to 0.03)	0.27	0.00 (−0.03 to 0.03)	0.99	−0.04 (−0.06 to 0.02)	<0.001
Triglyceride (mmol/L)	−0.09 (−0.17 to 0.02)	0.01	−0.20 (−0.31 to 0.09)	<0.001	−0.18 (−0.34 to 0.03)	0.02	0.08 (−0.06 to 0.22)	0.25
Fat mass index (kg/m^5^)	−0.18 (−0.25 to 0.10)	<0.0001	−0.19 (−0.30 to 0.07)	0.001	−0.32 (−0.47 to 0.16)	<0.0001	−0.05 (−0.19 to 0.08)	0.45

All models adjust for age, sex, month, an interaction between ethnic group and 25(OH)D_2_ or 25(OH)D_3_ and school (random effect).

P values for interaction between ethnicity and 25(OH)D_2_ were: insulin p=0.001, HOMA IR p=0.003, glucose p=0.33, HbA1c p=0.86, triglyceride p=0.04, fat mass index p=0.17.

P values for interaction between ethnicity and 25(OH)D_3_ were: insulin p=0.005, HOMA IR p=0.006, glucose p=0.96, HbA1c p<0.001, triglyceride p=0.002, fat mass index p=0.02.

NB: The total number includes the ‘other’ ethnic group which is not presented here.

HOMA IR, homoeostasis model assessment-insulin resistance; 25(OH)D_2_, 25-hydroxyvitamin D.

### Ethnic differences in vitamin D: potential contributions to ethnic differences in type 2 diabetes risk markers

To examine the potential contribution of 25(OH)D to explaining ethnic differences in fasting insulin and insulin resistance, the effect of adjustment for 25(OH)D on ethnic differences in fasting insulin and HOMA-IR between South Asians and White Europeans and separately between black African Caribbeans and white Europeans was examined (online supplemental table 4). The additional adjustment for total 25(OH)D reduced the higher levels of fasting insulin and HOMA-IR among both South Asian and black African Caribbean children (from 30.6% higher fasting insulin in South Asians to 17.8% higher once 25(OH)D was included in the models (a 42% relative reduction), and from 22.5% higher fasting insulin in black African Caribbean to 12.8% higher once 25(OH)D was included in the models) (a 43% relative reduction); very similar patterns were observed for HOMA-IR.

## Discussion

In this report, we report associations between 25(OH)D concentrations and risk markers for type 2 diabetes in a multiethnic population of white European, South Asian and black African Caribbean children and the potential contribution of ethnic differences in 25(OH)D to explaining the higher levels of insulin resistance observed in South Asian and black African Caribbean children. Strong inverse associations between total 25(OH)D and fasting insulin, HOMA-IR, fasting glucose and adiposity were observed in the whole study population and in each of the three specific ethnic groups, with no strong evidence of heterogeneity in different ethnic groups. Very similar patterns of associations to those for total 25(OH)D were observed for 25(OH)D_3,_ with somewhat less consistent associations observed for 25(OH)D_2_. Children of South Asian and black African Caribbean origins had markedly lower total 25(OH)D and 25(OH)D_3_ concentrations; adjustment for these differences reduced the higher levels of fasting insulin and insulin resistance among South Asian and black African Caribbean children by at least 40%.

### Previous studies

The inverse cross-sectional graded associations between circulating 25(OH)D concentrations, fasting insulin, insulin resistance and fasting glucose concentrations (which were independent of adiposity), are consistent with observational studies in both adults and children, which have previously demonstrated cross-sectional associations between 25(OH)D concentrations and insulin resistance.^30 31^ Inverse associations between 25(OH)D concentrations and incident type 2 diabetes have also been shown in large prospective studies in adults.^9 32^ The absence of any appreciable evidence of heterogeneity in the associations between 25(OH)D and insulin resistance between ethnic groups suggests that our findings are robust and are consistent with an earlier report which, although reporting weaker associations between 25(OH)D and glycaemia for non-Hispanic blacks than other ethnic groups, observed no ethnic heterogeneity in the associations between 25(OH)D and insulin resistance.^23^ These observational findings are also consistent with a meta-analysis of RCTs in adults which reported significant reductions in insulin resistance and glycaemia following supplementation with vitamin D.^17^ Furthermore, an RCT in children reported reductions in fasting glucose following vitamin D supplementation.^20^ Large RCTs of vitamin D supplementation in adults with pre-diabetes have generally shown around a 10%–15% reduction in type 2 diabetes incidence in this high-risk population,^21 33^ a statistically significant effect in a recent systematic review and meta-analysis of all published trials.^19^ Furthermore, post hoc analyses of randomised trials of vitamin D supplementation in participants who were vitamin D deficient at baseline, indicated potentially much larger relative reductions in the risk of type 2 diabetes of approximately 60%,^21^ similar to findings from a small RCT in adults with pre-diabetes and vitamin D deficiency.^34^ The significance of baseline vitamin status has also been highlighted in the limited number of supplemental trials in children; a trial of supplementation in vitamin D deficient children found significantly lower insulin resistance,^35^ compared with limited null findings reported from trials which generally included 25(OH)D replete children.^36^

Circulating 25(OH)D concentrations observed in this childhood population were slightly lower than those of the National Diet and Nutrition Survey (NDNS) conducted at approximately the same time (2008–2010).^5^ However, vitamin D concentrations were similar in NDNS children when compared with CHASE white European children only, suggesting that the lower average concentrations in the whole study sample may well have reflected the high prevalence of ethnic minority participants in the current study. The ethnic differences in 25(OH)D are consistent with patterns previously reported in UK adults, with low vitamin D status and a higher frequency of vitamin D deficiency in UK South Asian and black African Caribbeans.^6 37^ Differences in factors including skin pigmentation, body fatness patterns, time spent outdoors, patterns of skin coverage and dietary intakes may all have contributed to observed ethnic differences in 25(OH)D concentrations.^38^ The lower 25(OH)D concentrations in girls are also consistent with patterns reported in NDNS in children^5^ and with wider global patterns.^39^ Potential explanations suggested have included sex differences in fat mass and gender differences in patterns of clothing and outdoor sunlight exposures.^39 40^ The inverse associations between vitamin D status and adiposity levels have also previously been widely reported in both adults and children,^41 42^ and may be due to the reduction of the circulating bioavailability of vitamin D by sequestration of vitamin D into adipose tissue.^43^

### Strengths and limitations

The large study population was drawn from primary schools in three major UK cities, with school-based sampling designed to provide strong and balanced ethnic minority representation, especially for key ethnic groups at high risk of vitamin D deficiency and type 2 diabetes risk; the study design allowed for comparisons to be made on a within school basis. The primary school setting also allowed for early markers of type 2 diabetes risk to be measured and associations of these with type 2 diabetes; our findings highlighted the already present marked ethnic differences in insulin resistance and glycaemia in this young age group of the study participants, emphasising the potential scope for early type 2 diabetes prevention.^25^ A further strength is the inclusion of measurements of both 25(OH)D_2_ and 25(OH)D_3_ concentrations, included in few previous studies in children. Although the data are cross-sectional and observational, limiting causal inference, this design is nonetheless particularly appropriate for examining short-term associations between nutritional status and type 2 diabetes risk markers. However, we cannot exclude the possibility that results could be confounded by unmeasured factors that relate to both vitamin D and insulin resistance or other metabolic markers such as genetic factors and physical fitness. For analysis, children were categorised into broad ethnic groups of ‘South Asian’ and ‘black African Caribbean’. This was consistent with the key research questions addressed and allowed analyses to be conducted with reasonable statistical power and precision. Studies investigating heterogeneity in the vitamin D-type 2 diabetes risk associations between Bangladeshi and Pakistani children (eg) would have required considerably larger numbers of children in specific ethnic subgroups. The ethnic categories used are consistent with those recently recommended for use by the National Institutes for Health in the USA,^44^ although the use of the white European group as a reference group in the present report cannot be taken to imply that the characteristics of that group can be definitively considered ‘normal’.^45^ Although the fieldwork for the study was conducted in 2004–2007, it is likely that these sex and ethnic differences in vitamin D deficiency have persisted, in the absence of any new high-profile supplementation policy.^4^

### Implications

The results presented here show consistent graded inverse associations between circulating 25(OH)D and insulin resistance in all specific ethnic groups studied. Although the causal basis for the association between vitamin D concentrations and type 2 diabetes risk remains uncertain,^18^ recent trial evidence on the effects of vitamin D supplementation in both healthy adults^17^ and those with pre-diabetes^19^ which reported reduced type 2 risk markers and incidence, is suggestive of a causal relationship. Furthermore, most participants in vitamin D supplementation trials to date have been vitamin D replete and it is possible that people with low circulating vitamin D concentrations, could experience greater metabolic benefits from vitamin D supplementation.^18^ Our findings, emphasising the lower average vitamin D concentrations in girls and in children of ethnic minority origins, when placed in the context of experimental data from adults at high type 2 diabetes risk with low vitamin D concentrations,^34^ suggest that further trials of the effect of vitamin D supplementation on insulin resistance and glycaemia specifically among vitamin D depleted populations (potentially including UK South Asian and black African Caribbean children) should be prioritised. Apart from the likely benefits for skeletal health, such trials could usefully examine the effects of vitamin D supplementation on type 2 diabetes risks in adults and effects on insulin resistance and circulating glucose concentrations in children.

## Conclusion

The inverse cross-sectional associations between circulating vitamin D concentrations and insulin resistance observed in ethnically diverse children aged 9–10 years add to the growing observational and experimental evidence which suggest that increasing circulating vitamin D concentrations in children who are vitamin D deficient could reduce insulin resistance. Further evidence, particularly from robust RCTs of vitamin D supplementation could help to examine the validity of this strategy. Increasing vitamin D concentrations could be particularly relevant for children of South Asian and black African Caribbean origins who are more likely to be vitamin D deficient and are at increased long-term risks of type 2 diabetes compared with white European children.

## Data Availability

Data are available on reasonable request.
