# Supplementary material for: Cross-sectional study of the associations between circulating vitamin D concentrations and insulin resistance in children aged 9–10 years of South Asian, black African Caribbean and white European origins
Source: J Epidemiol Community Health. 2023 Dec 11;78(3):137–44. doi: 10.1136/jech-2023-220626 (PMC11045364; doi:10.1136/jech-2023-220626)
Supplement: Supplementary data [file jech-2023-220626supp001.pdf]

Supplementary analysis

Supplementary Figure 1: Distributions of total 25(OH)D (panel A), 25(OH)D_2 (panel B) and 25(OH)D_3. (panel C): by ethnic group

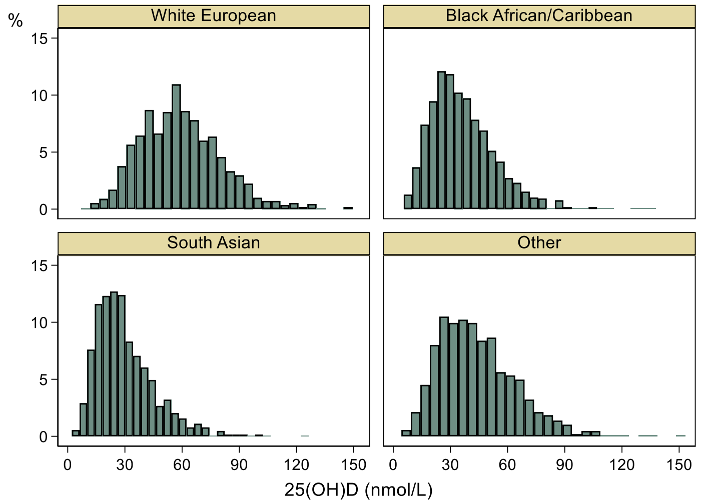

A

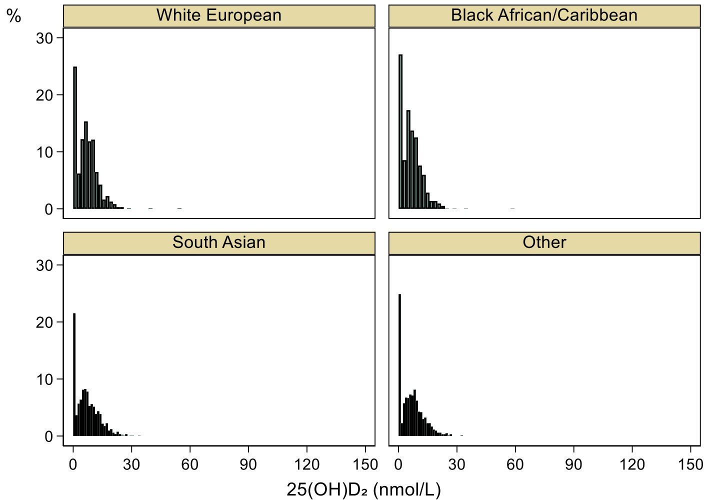

B

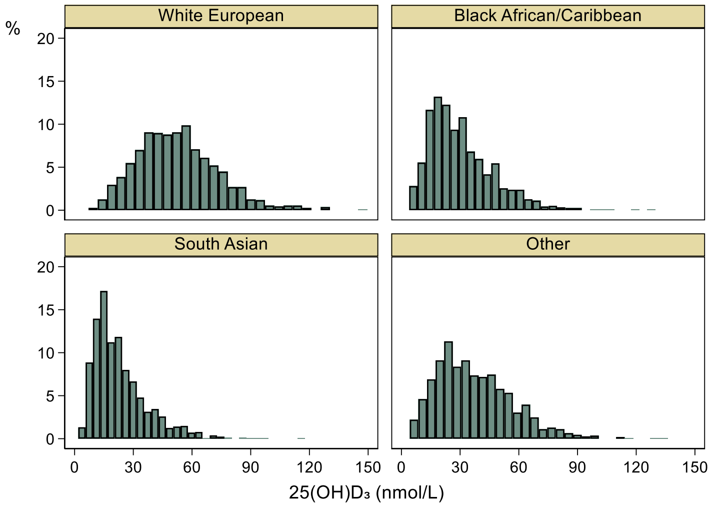
White European
Black African/Caribbean
South Asian
Other
%
20
15
10
5
0
0
30
60
90
120
150
25(OH)D₃ (nmol/L)

C

Supplementary Figure 2: Associations between fasting insulin and 25(OH)D_2 (panel A) and 25(OH)D_3 (panel B): by ethnic group

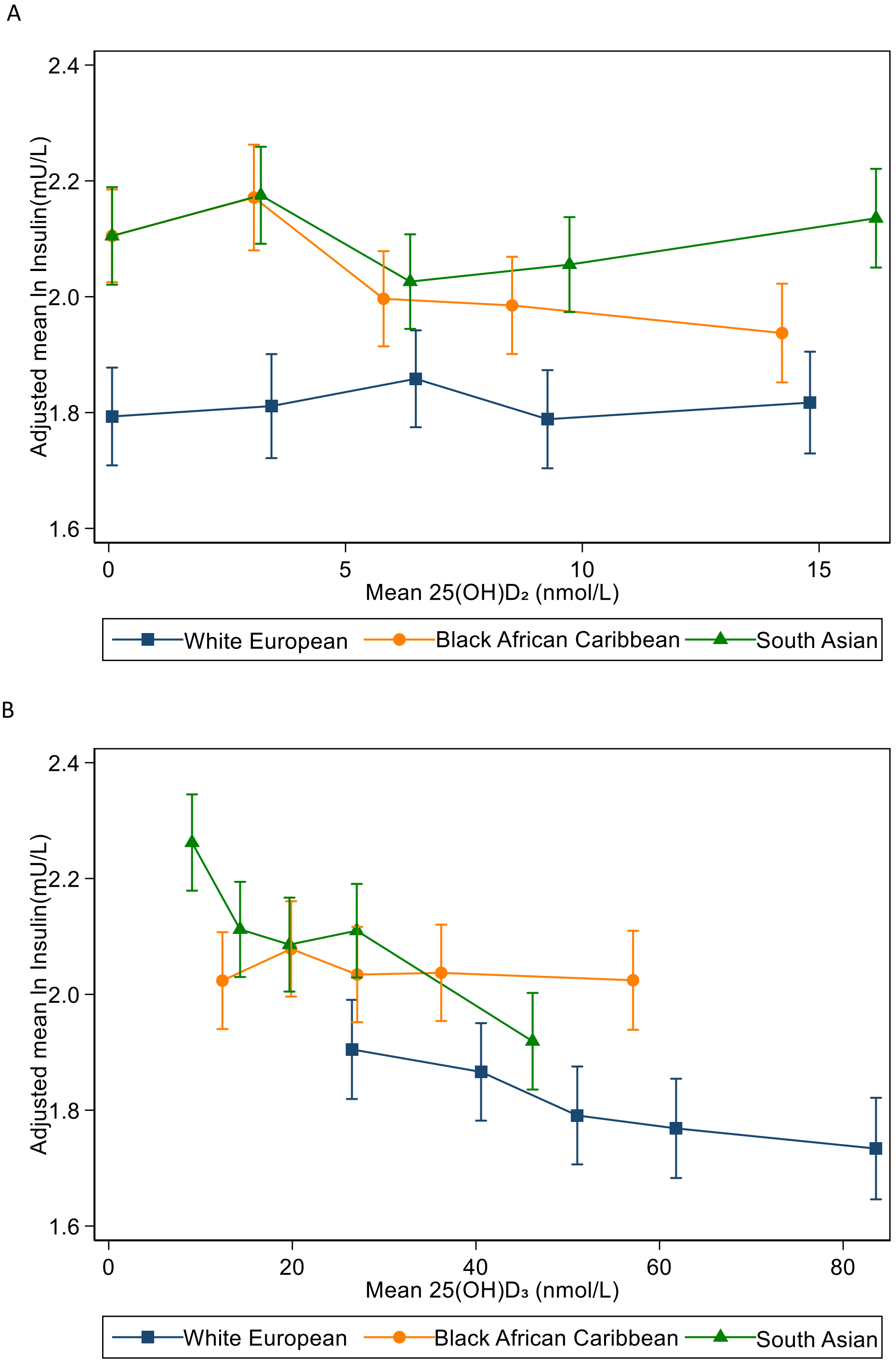

Supplementary Table 1: Population characteristics by quartiles of total 25(OH)D, 25(OH)D_2 and 25(OH)D_3 (nmol/L)

		Quartiles of total 25(OH)D (nmol/L)				
Range of vitamin 25(OH)D concentrations (nmol/L) per quartile		1<br>2.2 - 25.7	2<br>25.8 - 38.3	3<br>38.4 - 54.9	4<br>55 - 153.4	*p*(trend)
Age in years, mean (SE)		10.0 (0.01)	10.0 (0.01)	9.9 (0.01)	10.0 (0.01)	0.34*a*
Sex	% Girls	58	53	49	46	<0.001*b*
Socio-economic status	% Managerial & Professional	21	25	28	33	<0.001*b*
Ethnic group	% White European	3	12	27	54	<0.001*b*
	% Black African Caribbean	30	31	26	14	<0.001*b*
	% South Asian	50	33	19	8	<0.001*b*
Fat mass index (kg/m^5), mean (SE)		2.25 (0.03)	2.25 (0.03)	2.20 (0.03)	2.10 (0.02)	<0.001*a*
Cotinine ng/ml, mean (SE)		0.74 (0.30)	0.89 (0.16)	0.71 (0.05)	0.75 (0.04)	0.41*a*
		Quartiles of 25(OH)D_2 (nmol/L)				
Range of vitamin 25(OH)D_2 concentrations (nmol/L) per quartile		1<br>0.7 - 1.8	2<br>1.9 - 6.1	3<br>6.2 - 9.9	4<br>10.0 - 59.5	*p*(trend)
Age in years, mean (SE)		10.0 (0.01)	10.0 (0.01)	10.0 (0.01)	9.9 (0.01)	0.79*a*
Sex	% Girls	51	53	52	51	0.77*b*
Socio-economic status	% Managerial & Professional	30	26	25	27	0.08*b*
Ethnic group	% White European	24	22	26	24	0.29*b*
	% Black African Caribbean	27	28	26	21	0.001*b*
	% South Asian	25	28	25	31	0.01*b*
Fat mass index (kg/m^5), mean (SE)		2.17 (0.02)	2.19 (0.03)	2.16 (0.02)	2.28 (0.03)	0.004*a*
Cotinine ng/ml, mean (SE)		0.94 (0.31)	0.72 (0.05)	0.63 (0.04)	0.80 (0.13)	0.26*a*
		Quartiles of 25(OH)D_3 (nmol/L)				
Range of vitamin 25(OH)D_3 concentrations (nmol/L) per quartile		1<br>1.9 - 19.0	2<br>19.1 - 30.8	3<br>30.9 - 47.7	4<br>47.8 - 149.5	*p*(trend)
Age in years, mean (SE)		9.9 (0.01)	9.9 (0.01)	10.0 (0.01)	10.0 (0.01)	0.63*a*
Sex	% Girls	59	53	48	47	<0.001*b*
Socio-economic status	% Managerial & Professional	21	24	29	33	<0.001*b*
Ethnic group	% White European	3	11	29	54	<0.001*b*
	% Black African Caribbean	28	33	26	15	<0.001*b*
	% South Asian	53	33	17	7	<0.001*b*
Fat mass index (kg/m^5), mean (SE)		2.28 (0.03)	2.24 (0.03)	2.18 (0.03)	2.10 (0.02)	<0.001*a*
Cotinine ng/ml, mean (SE)		0.90 (0.32)	0.60 (0.05)	0.86 (0.11)	0.73 (0.04)	0.44*a*

a linear regression model; *b* Kruskal-Wallis tests for trend.

Supplementary Table 2: Associations between risk markers for type 2 diabetes and cardiovascular disease and total 25(OH)D, 25(OH)D_2 and 25(OH)D_3 (per IQR increase): by ethnic group

	Percentage difference in outcome per IQR increase in 25(OH)D (95% CI), p-value									
Outcome	All (n=4650)		White European (n = 1117)		South Asian (n = 1275)		Black African Caribbean (n = 1176)		Other ethnicities (n=1082)	
Insulin (mU/l)	-10.50 (-13.39, -7.52)	<0.0001	-9.14 (-13.80, -4.23)	<0.001	-15.62 (-21.15, -9.72)	<0.0001	-8.41 (-13.90, -2.57)	0.005	-10.29(-15.09, -5.22)	<0.001
HOMA Insulin resistance	-10.69 (-13.54, -7.74)	<0.0001	-9.49 (-14.09, -4.64)	<0.001	-15.42 (-20.91, -9.56)	<0.0001	-8.31 (-13.76, -2.52)	0.006	-10.78 (-15.50, -5.77)	<0.0001
Glucose (mmol/L)	-0.99 (-1.39, -0.59)	<0.0001	-0.84 (-1.49, -0.19)	0.01	-0.69 (-1.53, 0.15)	0.11	-0.96 (-1.72, -0.20)	0.01	-1.36 (-2.04, -0.69)	<0.0001
HbA1c (%)	-0.08 (-0.41, 0.25)	0.62	0.45 (-0.09, 0.98)	0.10	0.21 (-0.47, 0.89)	0.55	-1.03 (-1.65, -0.42)	0.001	-0.11 (-0.66, 0.45)	0.70
Triglyceride (mmol/l)	-0.18 (-2.17, 1.85)	0.86	-3.33 (-6.42, -0.14)	0.04	-0.93 (-4.97, 3.29)	0.66	3.17 (-0.69, 7.18)	0.11	1.28 (-2.10, 4.78)	0.46
Fat mass index (kg/m^5)	-3.88 (-5.80, -1.92)	<0.001	-4.20 (-7.28, -1.02)	0.01	-7.97 (-11.73, -4.05)	<0.0001	-0.79 (-4.53, 3.09)	0.68	-3.24 (-6.50, 0.12)	0.06
Outcome	Percentage difference in outcome per IQR increase in 25(OH)D_2 (95% CI), p-value									
Insulin (mU/l)	-3.13 (-5.92, -0.27)	0.03	1.24 (-4.20, 6.98)	0.66	-1.76 (-6.52, 3.23)	0.48	-11.60 (-16.35, -6.59)	<0.0001	-0.27 (-5.49, 5.23)	0.91
HOMA Insulin resistance	-3.17 (-5.92, -0.33)	0.03	1.02 (-4.35, 6.70)	0.72	-1.61 (-6.33, 3.35)	0.52	-10.90 (-15.64, -5.90)	<0.0001	-1.16 (-6.29, 4.24)	0.67
Glucose (mmol/L)	0.01 (-0.35, 0.38)	0.94	0.52 (-0.17, 1.22)	0.14	-0.13 (-0.75, 0.49)	0.68	-0.18 (-0.86, 0.51)	0.61	-0.11 (-0.78, 0.56)	0.75
HbA1c (%)	0.44 (0.15, 0.74)	0.003	0.50 (-0.06, 1.05)	0.08	0.40 (-0.09, 0.90)	0.11	0.23 (-0.33, 0.78)	0.42	0.66 (0.12, 1.20)	0.02
Triglyceride (mmol/l)	6.49 (4.62, 8.39)	<0.0001	7.92 (4.34, 11.62)	<0.0001	7.08 (3.88, 10.37)	<0.0001	2.20 (-1.19, 5.70)	0.21	8.64 (5.13, 12.27)	<0.0001
Fat mass index (kg/m^5)	1.97 (0.19, 3.78)	0.03	3.41 (-0.05, 6.99)	0.05	-1.12 (-4.09, 1.94)	0.47	1.35 (-2.03, 4.85)	0.44	5.07 (1.65, 8.61)	0.003
Outcome	Percentage difference in outcome per IQR increase in 25(OH)D_3 (95% CI), p-value									
Insulin (mU/l)	-10.22 (-13.20, -7.13)	<0.0001	-9.66 (-14.29, -4.77)	<0.001	-17.06 (-22.86, -10.82)	<0.0001	-4.82 (-10.65, 1.40)	0.13	-10.91 (-15.79, -5.76)	<0.0001
HOMA Insulin resistance	-10.40 (-13.35, -7.36)	<0.0001	-9.96 (-14.53, -5.14)	<0.0001	-16.90 (-22.66, -10.71)	<0.0001	-4.99 (-10.76, 1.15)	0.11	-11.17 (-15.99, -6.08)	<0.0001
Glucose (mmol/L)	-1.08 (-1.49, -0.66)	<0.0001	-1.00 (-1.65, -0.35)	0.003	-0.73 (-1.62, 0.18)	0.11	-0.99 (-1.76, -0.20)	0.01	-1.43 (-2.12, -0.74)	<0.0001
HbA1c (%)	-0.26 (-0.60, 0.07)	0.13	0.30 (-0.23, 0.83)	0.27	-0.01 (-0.73, 0.73)	0.99	-1.20 (-1.82, -0.56)	<0.001	-0.35 (-0.91, 0.22)	0.23
Triglyceride (mmol/l)	-2.62 (-4.62, -0.58)	0.01	-5.52 (-8.53, -2.40)	<0.001	-5.15 (-9.29, -0.82)	0.02	2.30 (-1.60, 6.37)	0.25	-1.43 (-4.79, 2.05)	0.42
Fat mass index (kg/m^5)	-4.94 (-6.89, -2.94)	<0.0001	-5.24 (-8.28, -2.10)	0.001	-8.64 (-12.63, -4.47)	<0.0001	-1.50 (-5.28, 2.43)	0.45	-5.05 (-8.31, -1.67	0.004

All models adjust for age, sex, month, an interaction between ethnic group and 25(OH)D or 25(OH)D_2 or 25(OH)D_3 and school (random effect)

25(OH)D IQR: 29.2nmol/L, 25(OH)D_2 IQR: 8.2nmol/L, 25(OH)D_3 IQR: 28.6nmol/L

Supplementary Table 3: Associations between risk markers for type 2 diabetes and cardiovascular disease and vitamin 25(OH)D (per nmol/L increase) with additional adjustment for fat mass index and socioeconomic status (NS-SEC)

Outcome (n = 4650)		Percentage difference in outcome per nmol/L increase in 25(OH)D (95% CI), p-value		Percentage difference in outcome per nmol/L increase in 25(OH)D_2 (95% CI), p-value		Percentage difference in outcome per nmol/L increase in 25(OH)D_3 (95% CI), p-value	
Insulin (mU/L)	Standard	-0.38 (-0.49, -0.27)	<0.0001	-0.39 (-0.74, -0.03)	0.03	-0.38 (-0.49, -0.26)	<0.0001
	Standard + FMI	-0.30 (-0.40, -0.20)	<0.0001	-0.59 (-0.91, -0.27)	<0.001	-0.27 (-0.37, -0.16)	<0.0001
	Standard + FMI + SES	-0.30 (-0.41, -0.20)	<0.0001	-0.59 (-0.91, -0.27)	<0.001	-0.27 (-0.38, -0.16)	<0.0001
HOMA Insulin resistance	Standard	-0.39 (-0.50, -0.28)	<0.0001	-0.39 (-0.74, -0.04)	0.03	-0.38 (-0.50, -0.27)	<0.0001
	Standard + FMI	-0.31 (-0.41, -0.21)	<0.0001	-0.59 (-0.91, -0.27)	<0.001	-0.28 (-0.38, -0.17)	<0.0001
	Standard + FMI + SES	-0.31 (-0.41, -0.21)	<0.0001	-0.59 (-0.91, -0.27)	<0.001	-0.28 (-0.39, -0.17)	<0.0001
Glucose (mmol/L)	Standard	-0.03 (-0.05, -0.02)	<0.0001	0.00 (-0.04, 0.05)	0.95	-0.04 (-0.05, -0.02)	<0.0001
	Standard + FMI	-0.03 (-0.05, -0.02)	<0.0001	0.00 (-0.05, 0.04)	0.92	-0.04 (-0.05, -0.02)	<0.0001
	Standard + FMI + SES	-0.03 (-0.05, -0.02)	<0.0001	0.00 (-0.05, 0.04)	0.94	-0.04 (-0.05, -0.02)	<0.0001
HbA1c (%)	Standard	0.00 (-0.01, 0.01)	0.62	0.05 (0.02, 0.09)	0.002	-0.01 (-0.02, 0.00)	0.12
	Standard + FMI	0.00 (-0.01, 0.01)	0.87	0.05 (0.01, 0.08)	0.01	-0.01 (-0.02, 0.01)	0.27
	Standard + FMI + SES	0.00 (-0.01, 0.01)	0.85	0.05 (0.01, 0.08)	0.01	-0.01 (-0.02, 0.00)	0.25
Triglyceride (mmol/L)	Standard	-0.01 (-0.08, 0.06)	0.85	0.76 (0.55, 0.98)	<0.0001	-0.09 (-0.17, -0.02)	0.01
	Standard + FMI	0.03 (-0.04, 0.09)	0.42	0.68 (0.47, 0.89)	<0.0001	-0.05 (-0.12, 0.02)	0.19
	Standard + FMI + SES	0.03 (-0.03, 0.10)	0.31	0.68 (0.48, 0.89)	<0.0001	-0.04 (-0.11, 0.03)	0.26

FMI = fat mass index, SES = socioeconomic status using NS-SEC classification

Standard adjustment is for age, sex, month, an interaction between ethnic group and 25(OH)D, and school (random effect)

Supplementary Table 4: Ethnic differences in insulin and HOMA insulin resistance: effect of adjustment for total 25(OH)D

Outcome (N = 4650)	Adjustment	Percentage difference (95% CI), p-value, % change from standard adjustment					
		South Asian - White European			Black African-Caribbean - White European		
Insulin (mU/L)	Standard	30.6 (24.0, 37.6)	<0.0001		22.5 (16.4, 29.0)	<0.0001	
	Standard + 25(OH)D	17.8 (10.9, 25.1)	<0.0001	-42%	12.8 (6.5, 19.3)	<0.0001	-43%
HOMA Insulin resistance	Standard	30.2 (23.7, 37.1)	<0.0001		22.0 (15.9, 28.3)	<0.0001	
	Standard + 25(OH)D	17.3 (10.5, 24.4)	<0.0001	-43%	12.1 (6.0, 18.6)	<0.0001	-45%

Standard adjustments are for age, sex, ethnic group, month and school (random effect)
